# Triclustering Model for Three-Dimensional Time-Series Gene Expression Data

**DOI:** 10.3390/ijms27125363

**Published:** 2026-06-14

**Authors:** Qiankun Liu, Mengyuan Zhu, Dongchao Ji, Libo Jiang

**Affiliations:** 1 College of Computer Science and Technology, Shandong University of Technology, Zibo 255000, China; dabaiyanglqk@163.com (Q.L.); myuanzhuu@163.com (M.Z.); 2 College of Life Sciences and Medicine, Shandong University of Technology, Zibo 255000, China; jidongchao@sdut.edu.cn

**Keywords:** cluster analysis, multivariate Gaussian mixture model, three-dimensional data, *Arabidopsis thaliana*

## Abstract

With the rapid advancement and cost reduction in high-throughput sequencing technologies, the accumulation of large-scale, three-dimensional gene expression data has surged. Consequently, effectively reducing the dimensionality of these complex datasets to extract critical biological information remains a significant challenge. Although various methods for identifying gene expression modules have been developed, most do not explicitly account for the multifactorial interactions among the temporal, spatial, and environmental dimensions. To address this limitation, we propose a novel three-dimensional triclustering technique based on a multivariate Gaussian mixture model (MVGMM) within a maximum likelihood framework. Specifically, our approach incorporates Legendre polynomials to model the temporal dynamics of gene expression and utilizes the Bayesian Information Criterion (BIC) to determine the optimal number of clusters. To further evaluate the model’s robustness against the high background noise typically present in empirical datasets (such as *Arabidopsis thaliana*), we conducted a rigorous sensitivity analysis by artificially injecting high-intensity Gaussian white noise into the simulated dataset. Despite severe noise interference, the global minimum of the BIC consistently remained at K = 6. Furthermore, the penalty term in the BIC successfully suppressed artificial cluster proliferation, preventing the model from fitting the noise as new functional modules. The MVGMM framework successfully recovered the predefined cluster structure in simulation studies and identified distinct expression modules in empirical *Arabidopsis thaliana* data. By jointly modeling temporal, spatial, and environmental variation, this study provides a statistical framework for exploring multidimensional gene expression patterns and may facilitate the identification of coordinated regulatory programs in complex biological systems.

## 1. Introduction

In recent years, rapid advances in biotechnology have established high-throughput sequencing as a primary approach for studying gene expression, ushering in a new era of genomics and bioinformatics [1]. The declining cost and growing maturity of these technologies have enabled researchers to generate massive volumes of gene expression data. Consequently, large-scale, multidimensional datasets—capturing variation across time points, tissues, and environmental conditions—are becoming increasingly prevalent [2], offering valuable opportunities for investigating the functions and regulatory mechanisms of biological systems. However, the complexity of such three-dimensional gene expression data [3] stems not only from their sheer size, but also from the intricate and diverse patterns of expression changes across different time points, tissues, environments, and even among individuals. This complexity greatly amplifies the challenge of data analysis. Therefore, extracting meaningful biological information from these enormous datasets has become an urgent scientific priority.

Given the inherent complexity of gene expression data and the challenges of analyzing them in three dimensions, an essential first step is to identify potentially relevant subspaces. Cluster analysis is a fundamental data mining technique that groups genes with similar expression patterns, thereby revealing potential functional relationships. The functional modules obtained from cluster analysis not only help infer relationships between candidate genes and transcription factors but also improve the annotation of functional genomes. Such clustering may help identify groups of genes with coordinated expression patterns and provide a useful framework for investigating potential regulatory relationships among genes. These expression modules can facilitate functional annotation and generate hypotheses regarding biological processes and regulatory mechanisms. Considering the importance of cluster analysis, a large number of methods have been developed for partitioning gene expression data into modules. Based on their statistical properties, these methods can be broadly categorized into three groups: (1) correlation- and distance-based approaches, such as K-means clustering [4] and hierarchical clustering [5]; (2) model-based approaches, including clustering based on Gaussian mixture models; and (3) machine learning-based methods, such as self-organizing maps [6]. In the context of three-dimensional data, subspace clustering is referred to as triclustering [7]. However, both the first and third categories generally assume that gene expression levels are independent of temporal or spatial scales, thereby neglecting the crucial temporal and spatial correlations of gene expression [8]. This limitation makes them poorly suited for effectively processing three-dimensional data. In contrast, model-based clustering determines the similarity of genes within a module by assessing how well their expression responses conform to a unified generative model—specifically, the degree to which the responses of any two genes fit the same generative model [9].

The field of genetics was among the first to adopt block clustering methodologies. Hartigan et al. initially introduced the concept of bidirectional clustering, termed “block clustering” [10]. Since Cheng et al. proposed a bidirectional clustering method for high-dimensional genetic data [11], such algorithms have been continuously developed and refined, finding widespread application in genetic analysis [12] and medicine [13]. Given the increasing complexity of data across spatial, temporal, and attribute dimensions, the development of multi-dimensional clustering techniques has become a critical research focus. A recent study introduced a three-way clustering technique for geographic big data that performs multi-dimensional clustering according to the spatial, temporal, and scale properties of the data and is applicable to various data types, including gene expression data [14]. This technique treats objects in three directions or dimensions as random variables; by iteratively adjusting the positions of the objects in these three dimensions, it identifies an optimal partitioning of the data in three-dimensional space—maximizing the similarity of data within each module while ensuring large differences between modules. However, this three-way clustering approach is fundamentally based on the principle of two-way clustering similarity. Its primary limitation is that it does not model correlations within and between different dimensions, which may reduce its ability to characterize complex dependencies in multidimensional gene expression data.

A time-dependent coding method for time series data has also been proposed. SplineCluster modeled the temporal dependence of gene expression data by fitting nonlinear spline basis functions to gene expression profiles, followed by Bayesian hierarchical clustering [15]. The Bayesian hierarchical clustering (BHC) algorithm treats Bayesian clustering as an approximation to the Dirichlet process (DP) model and iteratively merges clusters until the posterior probability of the merged model no longer exceeds that of the unmerged model [16,17,18]. Within the BHC framework, each cluster is parameterized using a Gaussian process (GP). However, because BHC employs a greedy merging strategy, it cannot fully capture the uncertainty inherent in the clustering process. For multidimensional gene expression data, statistical models should account for temporal, spatial, and environmental sources of variation, as these factors may contribute substantially to observed expression patterns. To address these limitations, this paper proposes a three-dimensional gene expression data clustering model based on a multivariate Gaussian mixture model (MVGMM). Specifically, we combine the MVGMM with Legendre polynomials [19] to flexibly fit the temporal dynamics of gene expression, and we employ the structured antedependence (SAD) model [20] to construct a parsimonious covariance matrix that captures interactions across time points, tissues, and environmental conditions. This framework models the correlations of gene expression across temporal, spatial, and environmental dimensions. Under the maximum likelihood framework, a hybrid algorithm integrating the Expectation–Maximization (EM) algorithm with simplex optimization is used to estimate the unknown model parameters. The optimal number of clusters is then determined using the Bayesian Information Criterion (BIC) [21], thereby enabling the three-dimensional clustering of gene expression data. In addition, a series of hypothesis tests [22] are proposed to examine the correlations of gene expression within and between modules, as well as across time, space, and environmental conditions.

To evaluate the performance of the proposed model for clustering three-dimensional gene expression data, we applied the algorithm to both simulated and published three-dimensional gene expression datasets. For the simulated dataset, we compared the obtained optimal number of clusters with the true, pre-specified number of clusters and conducted a series of hypothesis tests to identify specific expression patterns and multifactorial interactions. The model was subsequently applied to the *Arabidopsis thaliana* dataset, where the optimal number of clusters and the estimated model parameters were analyzed and evaluated. These results were further validated through hypothesis testing to confirm the accuracy of the detected expression patterns, thereby providing a statistical framework for exploring multidimensional gene expression patterns and their potential regulatory relationships.

## 2. Results

### 2.1. Application of the MVGMM to Simulated Data

To evaluate the performance of the proposed three-dimensional clustering method, we first assessed its statistical power and accuracy using computer simulations. The simulated dataset consisted of 500 genes measured across two tissues (tissue 1 and tissue 2) under two environmental conditions (control and treatment), yielding four distinct condition combinations (control–tissue 1, control–tissue 2, treatment–tissue 1, treatment–tissue 2). The time points for each combination were set as (1, 2, 3, 6, 12, 13, 15, 18, 22), (2, 7, 9, 10, 12, 13, 15), (1, 1.3, 1.6, 2, 2.4, 3, 4.5, 6.8, 7.2), and (4, 7, 8, 10, 11, 13, 16, 18, 22), respectively. Expression trajectories were fitted nonparametrically using Legendre polynomials, and the true number of gene modules was set to six.

#### 2.1.1. Determination of the Optimal Number of Clusters

**Figure 1:**
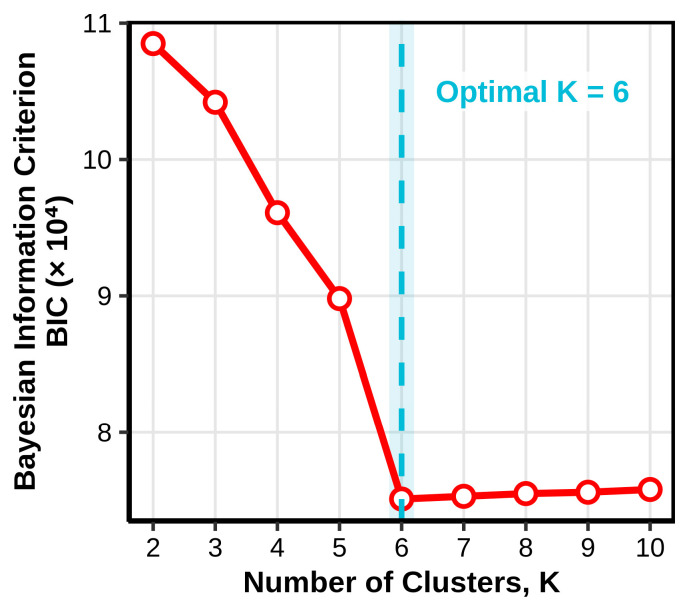
Determination of the optimal number of clusters for the simulated dataset using the Bayesian Information Criterion (BIC). BIC values were calculated for candidate cluster numbers ranging from K = 2 to K = 10. The x-axis indicates the number of clusters (K), and the y-axis shows the corresponding BIC value (×104). The BIC decreased with increasing cluster number and reached a minimum at K = 6 (blue dashed line), supporting the selection of six clusters for the simulated dataset.

The Bayesian Information Criterion (BIC) was computed for candidate cluster numbers ranging from 2 to 10 (Figure 1). The BIC value decreased sharply as the number of clusters increased from 2 to 5, indicating substantial improvements in model fit. At K = 6, the BIC reached its minimum and subsequently plateaued; further increases in cluster number no longer yielded meaningful reductions in the criterion. This demonstrates that the penalty term of the BIC effectively balances model complexity against goodness-of-fit. The model converged on K = 6, which exactly matches the predefined simulation setting, indicating that the BIC correctly selected the expected number of clusters in the simulated dataset.

#### 2.1.2. Statistical Validation of Identified Clusters

After determining the optimal number of clusters, we applied the six hypothesis tests described in Section 4.4 to validate the clustering results. First, pairwise comparisons between all clusters within each tissue–environment combination (Test 1, *p* < 0.05) revealed statistically significant differences between every pair of clusters (Supplementary Table S1), confirming that the six modules represent distinct expression patterns. Second, we tested for environmental responsiveness within each cluster for a given tissue (Test 2, *p* < 0.05); no significant differences between environments were detected (Supplementary Table S2), indicating that the simulated expression patterns were not modulated by environmental changes. Third, we tested for tissue specificity within each cluster under a fixed environment (Test 3, *p* < 0.05); again, no significant differences were observed (Supplementary Table S3). Fourth, tests for joint tissue–environment interactions (Test 4, *p* < 0.05) yielded no significant results (Supplementary Table S4). These outcomes support the distinctness of the six identified clusters and indicate consistency between the inferred clustering structure and the simulation design. Tests 5 and 6, which examine temporal and cross-condition correlations in the covariance matrix, further confirmed the adequacy of the SAD1 covariance parameterization (detailed results available upon request).

#### 2.1.3. Goodness-of-Fit and Residual Analysis

**Figure 2:**
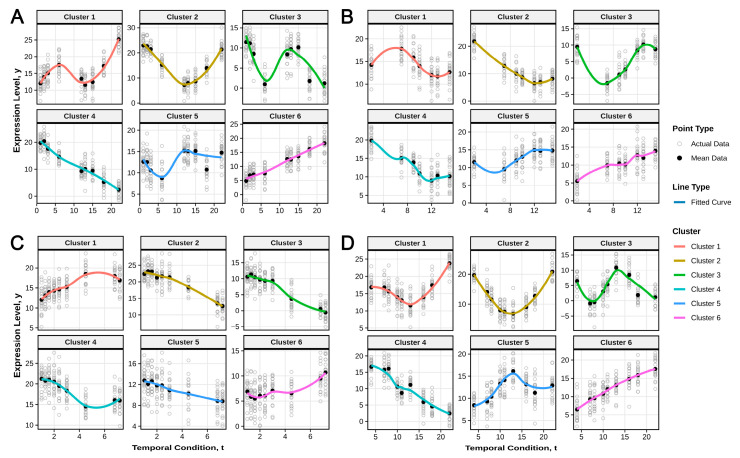
MVGMM-based fitting of gene expression trajectories under four simulated temporal conditions. Panels (**A**–**D**) show gene expression trajectories across TimeSets 1–4, respectively. Gray open circles represent the raw simulated expression values (Actual Data), and black solid circles represent the calculated empirical mean values at each time point (Mean Data). Colored curves represent the trajectories estimated by the MVGMM framework using Legendre polynomial functions. The fitted curves generally follow the observed mean expression patterns across clusters and temporal conditions. Residual distributions corresponding to the fitted models are presented in Supplementary Figure S1.

To further verify the accuracy of the model, the estimated parameters were used to reconstruct the mean expression trajectories for each cluster under each condition (Figure 2). The fitted curves closely tracked the empirical mean values across all time points, demonstrating a high degree of goodness-of-fit. To formally assess the quality of the fit, we computed the residuals—the differences between the observed gene expression levels and the fitted mean curves. As shown in Supplementary Figure S1, the residuals are symmetrically distributed around zero and closely approximate a normal distribution, suggesting that the model adequately captured the major expression trends in the simulated data without evidence of substantial systematic bias.

Sensitivity analysis under elevated noise. To address the concern that real-world datasets such as *Arabidopsis thaliana* often contain substantially higher noise levels than those present in the baseline simulation, we conducted a sensitivity analysis. Gaussian white noise with a standard deviation of 2.0 was artificially injected into the simulated dataset, and the MVGMM was re-run under identical algorithmic settings. Despite the elevated noise, the BIC correctly identified K = 6 as the optimal number of clusters, and the Adjusted Rand Index (ARI) between the noisy clustering result and the ground truth remained above 0.95. The variation of the BIC values under this severe noise interference, illustrating the effective suppression of artificial cluster proliferation, is detailed in Supplementary Figure S2. These results suggest that the MVGMM framework maintains stable cluster selection and clustering performance under elevated noise levels.

#### 2.1.4. Three-Dimensional Visualization

**Figure 3:**
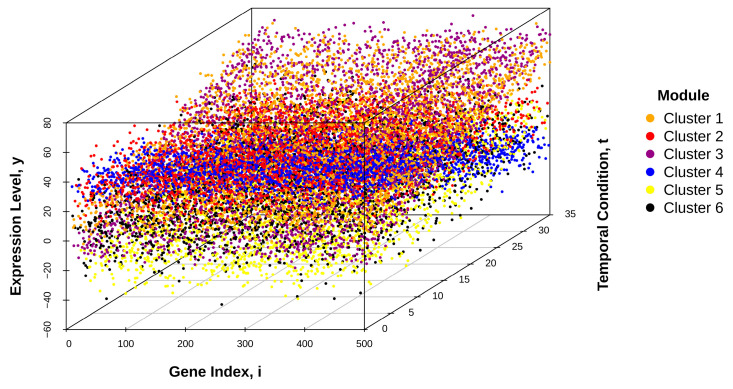
Three-dimensional spatial distribution of the simulated gene expression landscape. The 3D scatter plot visualizes the complex spatio-temporal dynamics of 500 simulated genes evaluated across 35 distinct temporal conditions. The x-axis represents the Gene Index (i), the y-axis represents the Temporal Condition (t), and the z-axis denotes the Expression Level (E). Data points are colored according to their cluster assignments obtained from the MVGMM framework. The visualization provides an overview of the clustering results and illustrates the distribution of gene expression patterns across the three-dimensional data space.

Based on the optimal clustering results, a three-dimensional scatter plot was generated (Figure 3), where the coordinates of each data point correspond to gene index, temporal condition, and expression level. Data points belonging to different modules are displayed in distinct colors. The spatial separation among clusters provides a visual representation of differences in expression patterns across modules and illustrates the clustering results obtained by the MVGMM framework.

#### 2.1.5. Performance Benchmarking

To quantitatively evaluate the computational performance and clustering accuracy of the proposed MVGMM, we benchmarked it against four established algorithms: traditional K-means, hierarchical clustering (HC), the Cheng and Church (CC) biclustering algorithm, and QUBIC. The Adjusted Rand Index (ARI) was used to measure the concordance between predicted module assignments and the predefined ground truth. As summarized in Table 1, the traditional and biclustering methods exhibited low computational cost (completing in less than one second) but suffered from substantial losses in accuracy (e.g., K-means ARI = 0.644) due to their inherent flattening of the three-dimensional data tensor, which do not model covariance structures across multiple dimensions. CC and QUBIC biclustering could not be directly evaluated by ARI because they produce overlapping or incomplete cluster assignments incompatible with the disjoint-module assumption of our framework. Both hierarchical clustering and MVGMM achieved a perfect ARI of 1.000 in this simulation. However, only MVGMM explicitly models the full spatiotemporal covariance structure, preserving the vast majority of the three-way interaction information that is lost by methods operating on flattened data matrices.

To evaluate the computational performance and clustering accuracy of the proposed MVGMM framework, we compared it with four commonly used clustering methods: traditional K-means, Hierarchical Clustering (HC), Cheng and Church (CC) biclustering, and QUBIC. The Adjusted Rand Index (ARI) was used to assess the agreement between inferred cluster assignments and the predefined simulation labels. As summarized in Table 1, K-means achieved lower clustering accuracy (ARI = 0.6444), whereas both HC and MVGMM achieved an ARI of 1.0000 under the simulated setting. CC and QUBIC could not be directly evaluated using ARI because they generate overlapping or incomplete cluster assignments that are not directly comparable with the disjoint cluster structure assumed in this study. While traditional clustering methods showed substantially lower computational cost, the MVGMM framework additionally models covariance structures across temporal, spatial, and environmental dimensions, enabling these dependencies to be incorporated into the clustering process.

### 2.2. Application of the MVGMM to the Arabidopsis Thaliana Dataset

The empirical *Arabidopsis thaliana* dataset analyzed in this study was obtained from a previously published experiment [23]. Briefly, seeds were surface-sterilized, stratified at 4 °C for three to five days, and sown on full-strength MS medium containing 1% sucrose and 0.8% agar. Plates were incubated at 22 °C under a 16 h light/8 h dark photoperiod. After four days, germinated seedlings were transferred to liquid culture. For RNA-seq, individual seedlings were placed in wells of a 24-well plate containing 1 mL of agar-free MS medium, and the plate was sealed with multi-well tape. For Ca^2+^ measurements, 30–50 seedlings per plate were transferred to a sterile 9 cm Petri dish containing approximately 25 mL of agar-free MS medium and sealed with porous tape. For soil-grown plants, seeds were lightly surface-disinfected, stratified for three to five days, and planted on soil. Plants were grown at 20 °C with 60% humidity under a 10 h light/14 h dark photoperiod for four to six weeks before assays were performed [23].

Gene expression data obtained from the above experiments were preprocessed prior to clustering. Genes with low variance were filtered out by comparing significant differences using multivariate ANOVA [24], yielding a final set of 1122 genes. The filtered data were then subjected to the three-dimensional MVGMM clustering pipeline.

**Figure 4:**
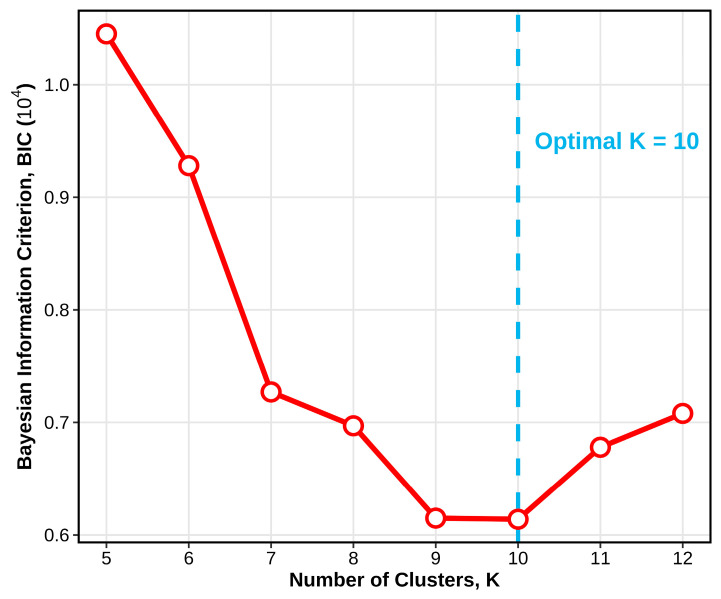
Determination of the optimal number of clusters for the empirical *Arabidopsis thaliana* dataset using the Bayesian Information Criterion (BIC). The line plot displays the calculated BIC values across a range of candidate cluster numbers (K = 5 to 12). The x-axis denotes the number of specified clusters (K), while the y-axis represents the model’s BIC penalty score (×104). The blue dashed vertical line marks the global minimum achieved at K = 10.

The BIC was computed for candidate cluster numbers K ranging from 5 to 12 (Figure 4). The BIC decreased as K increased from 5 to 9 and reached its minimum at K = 10. Beyond this point, the BIC increased with additional clusters, indicating that further increases in model complexity were not accompanied by sufficient improvements in model fit. Accordingly, K = 10 was selected as the optimal number of clusters for the *Arabidopsis thaliana* dataset.

The six hypothesis tests described in Section 4.4 were applied to validate the clustering structure. First, pairwise comparisons between all clusters within each tissue–environment combination (Test 1, *p* < 0.05) revealed statistically significant differences for every pair of clusters (Supplementary Table S5), confirming that the ten modules represent distinct expression patterns. Second, tests for environmental responsiveness within each cluster for a given tissue (Test 2, *p* < 0.05) detected no significant differences between environments (Supplementary Table S6). Third, tests for tissue specificity within each cluster under a fixed environment (Test 3, *p* < 0.05) likewise revealed no significant differences (Supplementary Table S7). Fourth, joint tissue–environment interaction tests (Test 4, *p* < 0.05) also produced no significant results (Supplementary Table S8). These results support the distinctness of the ten identified clusters. Significant differences were observed among clusters, whereas tests within clusters across tissues and environments did not reveal substantial heterogeneity. Tests 5 and 6, which evaluate temporal and cross-condition correlations within the SAD1 covariance structure, further supported the adequacy of the covariance parameterization (detailed results available upon request).

**Figure 5:**
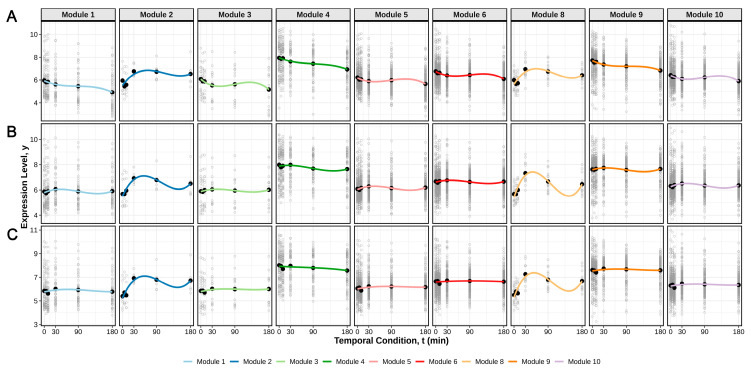
Performance evaluation of the MVGMM-based curve fitting for the empirical *Arabidopsis thaliana* dataset across three distinct experimental conditions. Panels (**A**–**C**) display the expression trajectories observed under Conditions 1–3, respectively. Gray open circles represent individual gene expression observations, while black solid circles indicate the corresponding empirical mean values at each time point. Colored curves represent the trajectories estimated by the MVGMM framework using Legendre polynomial functions.

To further validate the clustering results, the maximum likelihood parameter estimates were used to reconstruct the mean expression trajectories for the identified modules (Figure 5). Although the optimal number of clusters was determined to be K = 10, only nine modules are visibly represented in these specific temporal profiles because one cluster contained relatively few assigned genes under the displayed conditions. This reduction occurs because the EM algorithm’s penalty mechanics naturally suppress components (e.g., Module 7) that receive insufficient gene allocation under local conditions. For the manifested modules, the fitted curves closely tracked the empirical mean values across all time points and conditions, demonstrating a high degree of goodness-of-fit. The fitted curves generally followed the observed mean expression patterns across conditions. In addition, residuals were centered around zero with no obvious systematic trends, suggesting that the model adequately captured the major expression dynamics present in the data.

**Figure 6:**
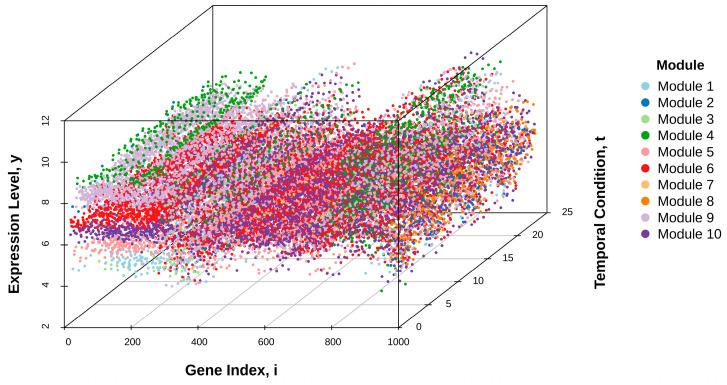
Three-dimensional spatial distribution of the empirical *Arabidopsis thaliana* gene expression clustering. The 3D scatter plot displays the expression profiles of 1122 filtered genes across the experimental conditions analyzed in this study. The x-axis represents the Gene Index (i), the y-axis represents the Temporal Condition (t), and the z-axis denotes the Expression Level (E). Data points are colored according to their cluster assignments obtained from the MVGMM framework. Differences in the spatial distribution of the colored clusters reflect variation in gene expression patterns among the identified modules.

Based on the clustering results, a three-dimensional scatter plot was generated (Figure 6), where the coordinates correspond to gene index, temporal condition, and expression level. Data points belonging to different modules are displayed in distinct colors. The visualization provides an overview of the distribution of genes across the identified clusters and illustrates variation in expression patterns within the three-dimensional data.

## 3. Discussion

In this study, we developed a multivariate Gaussian mixture model (MVGMM)-based framework for three-dimensional clustering of gene expression data. Traditional triclustering approaches are often derived from similarity-based clustering strategies and generally do not explicitly model correlations within and between different dimensions of the data. To address this limitation, the proposed framework integrates Legendre polynomials to flexibly fit the temporal dynamics of gene expression under the maximum likelihood framework, employs the structured antedependence (SAD) model to parsimoniously parameterize the high-dimensional covariance matrix, and uses the BIC to determine the optimal number of clusters. By jointly modeling temporal, spatial, and environmental variation, the MVGMM framework provides a statistical approach for clustering multidimensional gene expression data. Simulation analyses showed that the framework recovered the predefined clustering structure and captured characteristic expression patterns across multiple conditions.

The framework was first evaluated using simulated datasets, where the selected number of clusters was consistent with the predefined simulation setting. Application to the *Arabidopsis thaliana* dataset identified multiple expression modules with distinct temporal expression patterns. Sensitivity analyses conducted under increased noise levels indicated that cluster selection remained stable across the evaluated conditions. Performance benchmarking showed that the MVGMM achieved clustering accuracy comparable to hierarchical clustering in the simulation study while additionally providing estimates of cluster-specific mean trajectories and covariance structures. These features facilitate subsequent statistical inference and hypothesis testing within a unified probabilistic framework.

Like all statistical models, the MVGMM makes several assumptions about the data. Specifically, it assumes that (i) significant differences exist between any two clusters in a given tissue and environment; (ii) each cluster may exhibit distinct expression patterns across environments for a specific tissue; (iii) each cluster may show tissue-specific patterns under a given environment; (iv) each cluster may display joint tissue–environment interaction effects; (v) temporal autocorrelation exists within a given tissue–environment condition; and (vi) cross-condition temporal correlations are present between different tissues and environments [25]. Our hypothesis-testing framework systematically evaluates these assumptions, and the resulting statistical evidence supports the adequacy of the MVGMM framework for the datasets analyzed in this study.

The multidimensional gene expression patterns identified in this study may be related, in part, to higher-order chromatin organization. Nucleosomes occupy statistically defined positions along genomic DNA and influence local chromatin accessibility, thereby affecting transcriptional regulation and coordinated gene expression [26]. Consequently, some of the temporal and spatial expression modules identified by the MVGMM framework may reflect underlying chromatin states and regulatory environments. Although the present study does not directly measure nucleosome positioning or chromatin accessibility, the identified modules provide a useful basis for future investigations integrating transcriptomic and epigenomic information. Such analyses may help clarify the relationships between chromatin organization and multidimensional gene expression patterns.

The MVGMM framework can be extended in several directions. In addition to estimating mean expression trajectories, the model provides covariance parameters that may be useful for quantifying uncertainty in cluster assignment. The framework may also be applied to other multidimensional biological datasets, including epigenomic and proteomic measurements. Furthermore, alternative probability distributions could be incorporated when the Gaussian assumption is not appropriate for a given application. As multidimensional biological datasets continue to increase in size and complexity, statistical frameworks that jointly model multiple sources of variation may provide useful tools for exploring gene regulatory patterns and biological heterogeneity.

## 4. Materials and Methods

### 4.1. Simulation of Three-Dimensional Gene Expression Data

We assume that gene expression data from *C* tissues and *N* genes of a species are measured by microarray [27] or RNA-seq techniques [28] under *K* different environments and at *T* different time points. Let $y_{ickt}$ denote the expression level of gene *i* in tissue *c* under environment *k* at time point *t*, where *i* = 1, …, *N*, *c* = 1, …, *C*, *t* = 1, …, *T*, *k* = 1, …, *K*.

We assume that the *N* genes can be partitioned into *J* distinct modules, such that each gene belongs to exactly one module. For the expression data of all genes across multiple dimensions, the joint likelihood [29] can be expressed as:

(1) $$L(\theta )=\overset{N}{\underset{i=1}{\prod }}\sum_{j=1}^{J}\left[\pi_{j}f_{j}(y_{i};\mu_{j},\Sigma )\right]$$

where $\theta =(\pi_{j},\mu_{j},\Sigma )$ denotes the full set of model parameters; *π_j_* is the mixing proportion of module *j*, satisfying $\sum_{j=1}^{J}\pi_{j}=1$; $\mu_{j\;}$ is the mean vector of module *j*; Σ is the covariance matrix capturing variances and covariances across all three dimensions; and *f*(⋅) is the multivariate normal probability density function.

Because temporal gene expression patterns seldom follow a simple parametric form, we adopt a non-parametric approach using Legendre polynomials to fit the time-series data. We specifically chose Legendre polynomials over traditional B splines because of their strict orthogonality and superior numerical stability in high-dimensional Gaussian mixture frameworks. In our computational implementation, the temporal conditions are normalized to the standard interval [−1,1]. Within this domain, Legendre polynomials are completely orthogonal. When simulating three-dimensional gene expression data under the multivariate Gaussian mixture model (MVGMM), estimating the mean vectors and the high-dimensional SAD covariance matrix involves complex Expectation–Maximization (EM) iterations. If traditional B-splines were used, the design matrices often exhibit severe multicollinearity (ill-conditioning) when knot placement is dense or the data contain high levels of biological noise. This leads to numerical instability during matrix inversion. In contrast, the orthogonality of Legendre polynomials ensures that the polynomial coefficients are statistically independent, significantly enhancing the numerical stability of the EM algorithm and inherently preventing the model from overfitting high-noise features typical of empirical datasets such as *Arabidopsis thaliana*. This orthogonal design, combined with the BIC-based model selection described in Section 4.3, provides a robust mechanism to prevent overfitting even when the data exhibit substantial biological noise.

The Legendre polynomial of degree r is denoted by $P_{r}(x)$, where x∈[−1,1] is the normalized time variable.

(2) $$\begin{array}{c} P_{0}(x)=1,\;\;P_{1}(x)=x,\\ (r+1)P_{r+1}(x)=(2r+1)xP_{r}(x)-rP_{r-1}(x),\;\;\;\;r\geq 1. \end{array}$$

Based on the Legendre polynomials, the mean time-series vector of module j across tissues and environments can be expressed as:

(3) $$\mu_{jkc}(t)=\sum_{l=0}^{L_{jkc}}\beta_{jkc,l}P_{l}(x_{t})$$

where $L_{jkc\;}$ is the degree of the Legendre polynomial chosen to fit the mean time-series of module j in tissue c and environment *k*, and $\beta_{jkc,l}$ ($l=0,\ldots ,L_{jkc}$) are the unknown polynomial coefficients to be estimated.

### 4.2. Three-Dimensional Covariance Matrix Simulation

The covariance matrix [30] of gene expression data across the three dimensions of time, tissue, and environment can be expressed as:

(4) $$\Sigma =\left(\begin{array}{cccc} \Sigma_{11} & \Sigma_{12} & \ldots & \Sigma_{1,CK}\\ \Sigma_{21} & \Sigma_{22} & \ldots & \Sigma_{2,CK}\\ \vdots & \vdots & \ddots & \vdots \\ \Sigma_{CK,1} & \Sigma_{CK,2} & \ldots & \Sigma_{CK,CK} \end{array}\right)$$

In this matrix, each diagonal block $\Sigma_{ck,ck}$ represents the temporal covariance matrix for a specific tissue c and environment k, capturing the variance and serial correlation across time points within that single condition. The off-diagonal blocks $\Sigma_{ck,c^{\prime }k^{\prime }}$ encode covariances between different tissues, between different environments, or between different tissue–environment combinations; the full matrix is symmetric. Because this high-dimensional covariance matrix contains a large number of unknown parameters for three-dimensional data, direct unstructured estimation is computationally prohibitive and numerically unstable. To overcome this challenge, we employ the first-order structured antedependence (SAD1) model [20] to parameterize the covariance structure with substantially fewer parameters. This approach significantly improves both the accuracy of parameter estimation and the computational efficiency of the proposed method.

For a diagonal block (i.e., within a single tissue c and environment k), the SAD1 model assumes a constant innovation variance across all time points. The variance at time t can be expressed as:

(5) $$\sigma_{t}^{2}=\sigma^{2}\cdot \frac{1-\phi^{2t}}{1-\phi^{2}}$$

The covariance between two different time points $t_{1}$ and $t_{2}$ within the same tissue and environment is given by:

(6) $$\mathrm{Cov}\left(t_{1},t_{2}\right)=\sigma^{2}\cdot \phi^{\left|t_{2}-t_{1}\right|}\cdot \sqrt{\frac{1-\phi^{2t_{1}}}{1-\phi^{2t_{2}}}}\;(t_{2}\geq t_{1})$$

For off-diagonal blocks, the covariance between different tissues or different environments at the same time point t is modeled as:

(7) $${\mathrm{Cov}}_{ck,c^{\prime }k^{\prime }}(t)=\sigma_{c}\sigma_{c^{\prime }}\cdot \phi_{ck,c^{\prime }k^{\prime }}\cdot \sqrt{\frac{1-{\left(\phi_{c}\phi_{c^{\prime }}\right)}^{t}}{1-\phi_{c}^{2}}\cdot \frac{1-{\left(\phi_{c}\phi_{c^{\prime }}\right)}^{t}}{1-\phi_{c^{\prime }}^{2}}}$$

And the covariance between different time points $t_{1}$ and $t_{2}$ across different tissues or environments is given by:

(8) $${\mathrm{Cov}}_{ck,c^{\prime }k^{\prime }}(t_{1},t_{2})=\left\{\begin{array}{cc} \sigma_{c}\sigma_{c^{\prime }}\cdot \phi_{ck,c^{\prime }k^{\prime }}\cdot \frac{\phi_{c^{\prime }}^{t_{2}-t_{1}}-\phi_{c}^{t_{1}}\phi_{c^{\prime }}^{t_{2}}}{1-\phi_{c}\phi_{c^{\prime }}}\cdot \Gamma^{-1}, & t_{2}\geq t_{1}\\ \sigma_{c}\sigma_{c^{\prime }}\cdot \phi_{ck,c^{\prime }k^{\prime }}\cdot \frac{\phi_{c}^{t_{1}-t_{2}}-\phi_{c}^{t_{1}}\phi_{c^{\prime }}^{t_{2}}}{1-\phi_{c}\phi_{c^{\prime }}}\cdot \Gamma^{-1}, & t_{2}<t_{1} \end{array}\right.$$

where $\Gamma =\sqrt{\frac{1-\phi_{c}^{2t_{1}}}{1-\phi_{c}^{2}}\cdot \frac{1-\phi_{c^{\prime }}^{2t_{2}}}{1-\phi_{c^{\prime }}^{2}}}$. Through the parameters $\sigma^{2}$, ϕ, and $\phi_{ck,c^{\prime }k^{\prime }}$, the full three-dimensional covariance matrix in Equation (4) can be constructed parsimoniously, thereby enabling a comprehensive evaluation of both within-dimension and between-dimension correlations.

To initiate the numerical optimization of the SAD1 parameters in Equations (5)–(8), we assigned empirically motivated initial values to ensure stable convergence. Specifically, all innovation variances $\sigma^{2}$ were uniformly initialized to 1. The within-factor temporal autocorrelation parameters ϕ were initially set to 0.2, and the between-factor cross-correlation parameters $\phi_{ck,c^{\prime }k^{\prime }}$ were initialized to 0.5. These initial estimates were first refined using the Nelder–Mead simplex algorithm for up to 200 iterations before being passed into the main Expectation–Maximization (EM) framework.

### 4.3. Maximum Likelihood Estimation via the EM Algorithm

We estimate the model parameters—including the mean vectors, the covariance matrix, and the mixing proportions—within the maximum likelihood framework. Because the covariance matrix has a complex structure, closed-form analytical solutions for the covariance parameters are not available. We therefore employ a hybrid algorithm that combines the Expectation–Maximization (EM) algorithm with numerical optimization [31] to estimate the full set of unknown parameters in the likelihood function (1). Specifically, the EM algorithm is used to estimate the mixing proportions and the mean vector of each module; the Gaussian elimination method [32] is then applied to solve for the Legendre polynomial coefficients within the mean structure; and the Nelder–Mead simplex algorithm is employed to optimize the covariance parameters.

The EM algorithm proceeds by iterating between an expectation step (E-step) and a maximization step (M-step). In the E-step, the posterior probability that gene i belongs to module j is calculated based on the current parameter estimates:

(9) $$\omega_{ij}=\frac{\pi_{j}\cdot f(y_{i}|\mu_{j},\Sigma )}{\sum_{j^{\prime }=1}^{J}\pi_{j^{\prime }}\cdot f(y_{i}|\mu_{j^{\prime }},\Sigma )}$$

In the M-step, the mixing proportions are updated using these posterior probabilities:

(10) $$\pi_{j}=\frac{1}{N}\sum_{i=1}^{N}\omega_{ij}$$

The mean vector for each module is then re-estimated as a weighted average of the observed expression profiles:

(11) $$\mu_{j}=\frac{\sum_{i=1}^{N}\omega_{ij}y_{i}}{\sum_{i=1}^{N}\omega_{ij}}$$

To recover the Legendre polynomial coefficients that parameterize the time-dependent mean trajectories, we construct, for each tissue and environment, a system of linear equations based on the estimated mean vectors:

(12) $$L_{ck}\beta_{jkc}={\bar{y}}_{jkc}$$

where $L_{ck}$ is the design matrix of Legendre polynomials evaluated at the normalized time points, $\beta_{jkc}$ is the vector of unknown polynomial coefficients for module j in tissue c and environment k, and ${\overset{ˉ}{y}}_{jkc}$ is the corresponding segment of the weighted mean vector obtained from Equation (11). This linear system is solved efficiently using Gaussian elimination [32].

With the mixing proportions and mean parameters updated, the covariance parameters are then optimized by substituting the current estimates into the likelihood function (1) and applying the Nelder–Mead simplex algorithm. The E- and M-steps are iterated until convergence, which we define as the point at which the relative change in the log-likelihood falls below $10^{-5}$ or the maximum number of iterations (200) is reached.

Once the EM algorithm has converged for a given number of modules J, the optimal number of clusters is determined using the Bayesian Information Criterion (BIC) [21]:

(13) $$BIC(J)=2logL({\hat{\theta }}_{J})+p_{J}log(N\cdot n_{ts})$$

where $logL({\hat{\theta }}_{J})$ is the maximized log-likelihood for the model with J modules, $p_{J}$ is the total number of free parameters, N is the number of genes, and $n_{\mathrm{ts}}$ is the total number of time–tissue–environment observations per gene. The BIC balances model fit against model complexity, penalizing overly parameterized models. We compute the BIC across a range of candidate J values and select the J that yields the minimum BIC as the optimal number of clusters. This model selection strategy provides an inherent safeguard against overfitting, as it favors parsimonious models that adequately describe the data without capturing spurious noise-driven patterns, offering a computationally efficient alternative to resampling-based consensus clustering approaches [33]. All algorithmic simulations, statistical analyses, and data visualizations in this study were implemented using R software (version 4.3.3; R Foundation for Statistical Computing, Vienna, Austria). Core computations and data processing were performed utilizing packages including ggplot2, dplyr, MASS, and biclust.

### 4.4. Hypothesis Testing for Cluster Specificity

After completing the maximum likelihood estimation of all parameters and determining the optimal number of clusters, we propose a series of hypothesis tests to examine the statistical significance of the identified expression patterns. These tests assess differences in gene expression patterns between modules, across tissues, and across environments, as well as the temporal correlation structure within the covariance matrix. A significance level of p<0.05 is adopted throughout.

Test 1: Between-cluster differentiation in a specific tissue and environment.

To determine whether any two clusters $j_{1}$ and $j_{2}$ exhibit significantly different gene expression patterns in a particular tissue c and environment k, we test:

(14) $$H_{0}:\mu_{j_{1}kc}(t)=\mu_{j_{2}kc}(t)\;\mathrm{vs}.\;H_{1}:\mu_{j_{1}kc}(t)\neq \mu_{j_{2}kc}(t)$$

where $\mu_{jkc}(t)$ denotes the mean expression trajectory of module j in tissue c and environment k over time t. Rejection of $H_{0}$ indicates that the two clusters represent distinct expression patterns under the given conditions. Pairwise comparisons are performed across all clusters; a set of clusters in which all pairwise tests are significant supports the adequacy of the chosen number of clusters for that tissue–environment combination.

Test 2: Environmental responsiveness of a cluster in a specific tissue.

For a given cluster j and tissue c, we test whether the expression pattern differs across two environments $k_{1}$ and $k_{2}$:

(15) $$H_{0}:\mu_{jck_{1}}(t)=\mu_{jck_{2}}(t)\;\mathrm{vs}.\;H_{1}:\mu_{jck_{1}}(t)\neq \mu_{jck_{2}}(t)$$

Rejection of $H_{0}$ suggests that the expression of genes in this cluster is modulated by environmental conditions in tissue c. This test enables the identification of clusters that are specifically responsive to environmental changes within a given tissue.

Test 3: Tissue specificity of a cluster under a fixed environment

For a given cluster j and environment k, we test whether the expression pattern differs between two tissues $c_{1}$ and $c_{2}$:

(16) $$H_{0}:\mu_{jc_{1}k}(t)=\mu_{jc_{2}k}(t)\;\mathrm{vs}.\;H_{1}:\mu_{jc_{1}k}(t)\neq \mu_{jc_{2}k}(t)$$

Rejection of $H_{0}$ indicates that the cluster exhibits a tissue-specific expression pattern under environment k. This test facilitates the screening of clusters with spatially restricted expression profiles.

Test 4: Joint tissue–environment interaction effect for a cluster.

To detect whether a cluster displays significant tissue–environment interaction, we test the composite null hypothesis that the expression pattern is identical across all combinations of tissues and environments:

(17) $$H_{0}:\mu_{jc_{1}k_{1}}\left(t\right)=\mu_{jc_{1}k_{2}}\left(t\right)=\mu_{jc_{2}k_{1}}\left(t\right)=\mu_{jc_{2}k_{2}}\left(t\right)\;\mathrm{vs}.\;H_{1}:\mathrm{at}\;\mathrm{least}\;\mathrm{one}\;\mathrm{equality}\;\mathrm{does}\;\mathrm{not}\;\mathrm{hold}$$

Rejection of $H_{0}$ implies that the cluster exhibits both tissue-specific and environment-responsive expression patterns simultaneously.

Test 5: Within-condition temporal correlation of gene expression.

Using the temporal autocorrelation parameters ϕ estimated in the SAD1 covariance structure, we test whether gene expression in a specific tissue c and environment k shows significant temporal dependence:

(18) $$H_{0}:\phi_{ck}=0\;\mathrm{vs}.\;H_{1}:\phi_{ck}\neq 0$$

Under $H_{0}$, gene expression is independent across time points, implying a lack of serial correlation. Rejection of $H_{0}$ confirms that the expression data possess significant temporal structure, which justifies the use of time-series modeling approaches such as the Legendre polynomial fitting employed in our framework.

Test 6: Cross-condition temporal correlation.

Finally, we test whether gene expression in different tissues or environments is temporally correlated, using the between-factor correlation parameters $\phi_{ck,c^{\prime }k^{\prime }}$ from the SAD1 model:

(19) $$H_{0}:\phi_{ck,c^{\prime }k^{\prime }}=0\;\mathrm{vs}.\;H_{1}:\phi_{ck,c^{\prime }k^{\prime }}\neq 0$$

Under $H_{0}$, gene expression in tissue c and environment k is temporally independent of that in tissue $c^{\prime }$ and environment $k^{\prime }$. Rejection of $H_{0}$ indicates significant cross-condition temporal coordination of gene expression.

## 5. Conclusions

In this study, we developed a novel three-dimensional triclustering framework for gene expression data based on the multivariate Gaussian mixture model (MVGMM). By integrating Legendre polynomials for flexible time-series fitting, the structured antedependence (SAD) model for parsimonious covariance estimation, and the Bayesian Information Criterion (BIC) for optimal model selection, our approach successfully captures the complex interactions among temporal, spatial, and environmental dimensions of gene expression.

Both simulated and empirical evaluations demonstrated the effectiveness of the framework. On simulated data, the MVGMM accurately recovered the true number of clusters (K = 6) and achieved an Adjusted Rand Index of 1.000, outperforming traditional clustering and biclustering methods that sacrifice accuracy by ignoring spatiotemporal covariance structure. Sensitivity analysis confirmed that the model remains robust under elevated noise levels characteristic of real-world transcriptomic data. Application to the *Arabidopsis thaliana* dataset identified ten biologically distinct gene modules, revealing coordinated expression patterns that likely reflect shared regulatory mechanisms.

Critically, the gene expression clustering patterns detected by our framework do not arise in isolation; they are deeply connected to the physical organization of the genome. As demonstrated by Teif et al. [26], nucleosomes occupy statistically well-defined, base-pair-specific positions on genomic DNA, and the energetics of histone–DNA interactions dictate local chromatin accessibility. These precisely positioned nucleosomes create alternating domains of open and closed chromatin that physically coordinate the co-regulation of adjacent genes. Consequently, a substantial fraction of the temporal and spatial clustering effects identified by our MVGMM framework can be understood as emergent properties of this underlying nucleosome landscape. Our clustering results may therefore serve as a bridge connecting the static, sequence-dependent positioning of nucleosomes to the dynamic, condition-dependent patterns of gene co-expression.

The MVGMM framework provides a robust, statistically principled, and versatile computational tool for mining multi-dimensional genomic data. Future work may extend this approach in several directions: incorporating alternative distributions such as the Student-t process [34] to handle data types with heavier-tailed noise characteristics; adapting the framework for other three-dimensional omics data, including epigenomic and proteomic measurements; and developing computationally accelerated implementations to facilitate the analysis of increasingly large-scale genomic datasets.

## Figures and Tables

**Table 1:** Comprehensive Performance Benchmarking of Clustering Algorithms on the Simulated Dataset.

Algorithm	Computation Time (s)	Memory (MB)	Clustering Accuracy (ARI)	Spatiotemporal Covariance
Traditional K-means	~0.005	~0.20	0.644	Ignored
Hierarchical Clustering	~0.137	~4.90	1.000	Ignored
CC Biclustering	~0.012	~0.30	N/A	Ignored
QUBIC Biclustering	~0.672	~0.40	N/A	Ignored
MVGMM	~2700.0	~125.0	1.000	~99.25

Note: ARI: Adjusted Rand Index; CC: Cheng and Church; MVGMM: Multivariate Gaussian Mixture Model. “N/A” (Not Applicable) indicates that the ARI could not be calculated because the CC and QUBIC algorithms produce overlapping or incomplete cluster assignments, which are incompatible with the disjoint-module evaluation metric used here.

## Data Availability

The source code and simulated datasets for the 3D triclustering model are publicly available in the GitHub repository: https://github.com/DBYLqk/3D-Gene-Clustering. (accessed on 11 June 2026) The empirical *Arabidopsis thaliana* dataset processed for this study is also provided within the repository’s data folder. Other data related to this study are available from the corresponding author upon reasonable request.

## Supplementary Materials

The following supporting information can be downloaded at https://www.mdpi.com/article/10.3390/ijms27125363/s1.
