# Supplementary material for: Triclustering Model for Three-Dimensional Time-Series Gene Expression Data
Source: Int J Mol Sci. 2026 Jun 14;27(12):5363. doi: 10.3390/ijms27125363 (PMC13299315; doi:10.3390/ijms27125363)
Supplement: Supplementary file 1 [file ijms-27-05363-s001.zip › Supplementary Figure.pdf]

Figure S1

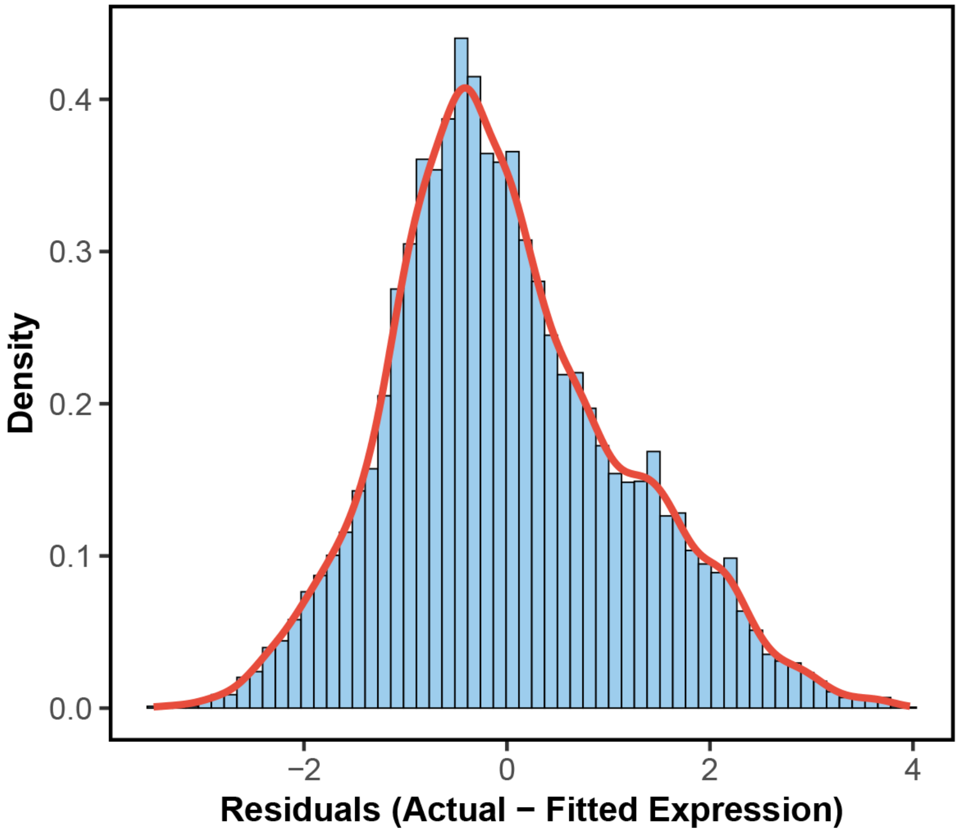

Figure S1. Distribution of residuals for the MVGMM-based three-dimensional gene expression clustering. The histogram displays the distribution of the differences (residuals) between the actual empirical gene expression levels and the mean curves fitted by the multivariate Gaussian mixture model combined with Legendre polynomials. The light blue bars represent the frequency distribution of the residuals, while the overlaid red curve represents the kernel density estimate. The symmetric, bell-shaped distribution closely centered around zero indicates that the residual errors approximate a normal distribution. This confirms that the model effectively captures the core mean trends of the complex spatiotemporal data without introducing significant systematic bias.

Figure S2

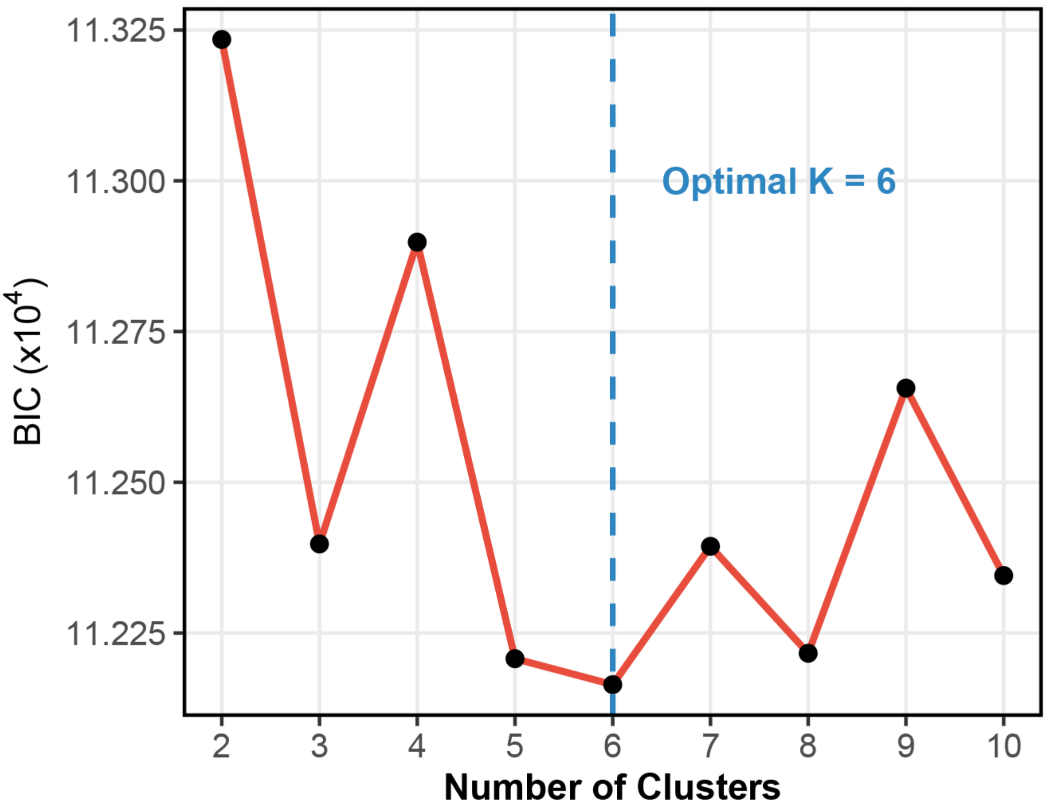

Figure S2. Sensitivity analysis of the clustering model under high-intensity background noise. The line graph illustrates the variation of the Bayesian Information Criterion (BIC) values as a function of the number of candidate clusters (ranging from 2 to 10) on the simulated dataset. To evaluate the model's robustness, high-intensity Gaussian white noise (standard deviation σ = 2.0) was artificially injected into the data prior to the Expectation-Maximization (EM) iterations. The x-axis represents the prescribed number of clusters, and the y-axis represents the corresponding BIC values scaled by 10^4. The blue vertical dashed line highlights the global minimum of the BIC curve, which accurately aligns with the optimal cluster number (K = 6). Although the severe noise interference induces visible local fluctuations (e.g., secondary peaks at K = 4 and K = 9), the BIC penalty term effectively suppresses artificial cluster proliferation. This demonstrates that the MVGMM framework firmly prevents overfitting and consistently converges to the true underlying structure even in highly noisy biological environments.
